# Activin in acute pancreatitis: Potential risk-stratifying marker and novel therapeutic target

**DOI:** 10.1038/s41598-017-13000-3

**Published:** 2017-10-06

**Authors:** Jonas J. Staudacher, Cemal Yazici, Timothy Carroll, Jessica Bauer, Jingbo Pang, Nancy Krett, Yinglin Xia, Annette Wilson, Georgios Papachristou, Andrea Dirmeier, Claudia Kunst, David C. Whitcomb, Giamila Fantuzzi, Barbara Jung

**Affiliations:** 1 Department of Medicine, Division of Gastroenterology and Hepatology, University of Illinois at Chicago, Chicago, IL USA; 2 Department of Kinesiology and Nutrition, University of Illinois at Chicago, Chicago, IL USA; 3 Department of Medicine, Division of Gastroenterology, Hepatology and Nutrition, University of Pittsburgh, Pittsburgh, PA USA; 4 Division of Gastroenterology and Hepatology, Veterans Affairs Health System Pittsburgh, Pittsburgh, PA USA; 5 Department of Internal Medicine I, University Medical Center Regensburg, Regensburg, Germany

## Abstract

Acute Pancreatitis is a substantial health care challenge with increasing incidence. Patients who develop severe disease have considerable mortality. Currently, no reliable predictive marker to identify patients at risk for severe disease exists. Treatment is limited to rehydration and supporting care suggesting an urgent need to develop novel approaches to improve standard care. Activin is a critical modulator of inflammatory responses, but has not been assessed in pancreatitis. Here, we demonstrate that serum activin is elevated and strongly correlates with disease severity in two established murine models of acute pancreatitis induced by either cerulein or IL-12 + IL-18. Furthermore, in mice, inhibition of activin conveys survival benefits in pancreatitis. In addition, serum activin levels were measured from a retrospective clinical cohort of pancreatitis patients and high activin levels in patients at admission are predictive of worse outcomes, indicated by longer overall hospital and intensive care unit stays. Taken together, activin is a novel candidate as a clinical marker to identify those acute pancreatitis patients with severe disease who would benefit from aggressive treatment and activin may be a therapeutic target in severe acute pancreatitis.

## Introduction

Acute Pancreatitis (AP) is the sterile inflammation of the pancreas in response to various insults. The incidence of acute pancreatitis is rising in the developed world^1^. With an incidence of 58 cases per 100,000 inhabitants, AP is the most common gastroenterological cause of hospitalization in the United States^2^. In most cases, AP is self-limiting and resolves in the first week after symptom onset. However, a substantial subset of patients develops local complications or organ failure. Severe pancreatitis, defined by current guidelines^3,4^ as persistent organ failure for more than 48 hours, is associated with high mortality rates in the range of 15–20%^5^, partly due to limited therapeutic options^3^.

Activin, a TGF-β superfamily member, is a cytokine with multiple context specific functions. After ligand binding to its type II receptors, activin type I receptors are activated through dimerization and phosphorylation^6^, leading to the activation of canonical SMAD-dependent^7^ and non-canonical SMAD-independent pathways^7,8^. Two major physiologic inhibitors are known, the competitive antagonist inhibin^9^ and the specific ligand trap follistatin^10,11^. First described as a reproductive hormone upstream of follicle-stimulating hormone (FSH)^12^, subsequent studies showed substantial roles in such diverse contexts as embryogenesis^13^, cancer^8,14,15^, and inflammation^16^.

In inflammation, activin has been reported to have both pro- and anti-inflammatory functions *ex vivo*, resulting in either up-or down-regulation of a number of key inflammatory cytokines, such as IL-6, IL-1-β or IL10 in a spectrum of human and murine cell types^17–20^. *In vivo*, activin’s reported actions are primarily pro-inflammatory. Systemic levels increase very early in the inflammatory response to LPS even before tumor necrosis factor (TNF)^21^. Furthermore, activin plays a central role in such diverse inflammatory conditions as an experimental model of inflammatory bowel disease (IBD)^22^, asthma^23^ and viral infections^24^. Given the substantial role activin plays in these conditions, we hypothesized that activin may be upregulated in AP, and constitute a potential marker of disease severity or a novel therapeutic target.

Treatment of severe AP is a clinical challenge and currently limited to supportive care with pain control and aggressive hydration therapy^3^. Despite studies demonstrating a possible clinical benefit when started early^25^, the use of broad spectrum antibiotics remains controversial and is not recommended in the US^3^. Despite the identification of several candidate biomarkers purported to associate with severe acute pancreatitis^26,27^, risk stratification of pancreatitis patients has proven difficult, and a simple and reliable method to identify patients at risk for developing severe AP is lacking^28^. Such a tool would have the potential to reduce overall health care cost through reduction of hospitalization and increase survival through early aggressive treatment in patient populations at risk while allowing for early discharge of patients with mild disease.

Here, we present the first study assessing activin as a potential marker and/or therapeutic target in AP.

## Results

### Activin is increased in mild AP *in vivo* and correlates with markers of disease severity

**Figure 1:**
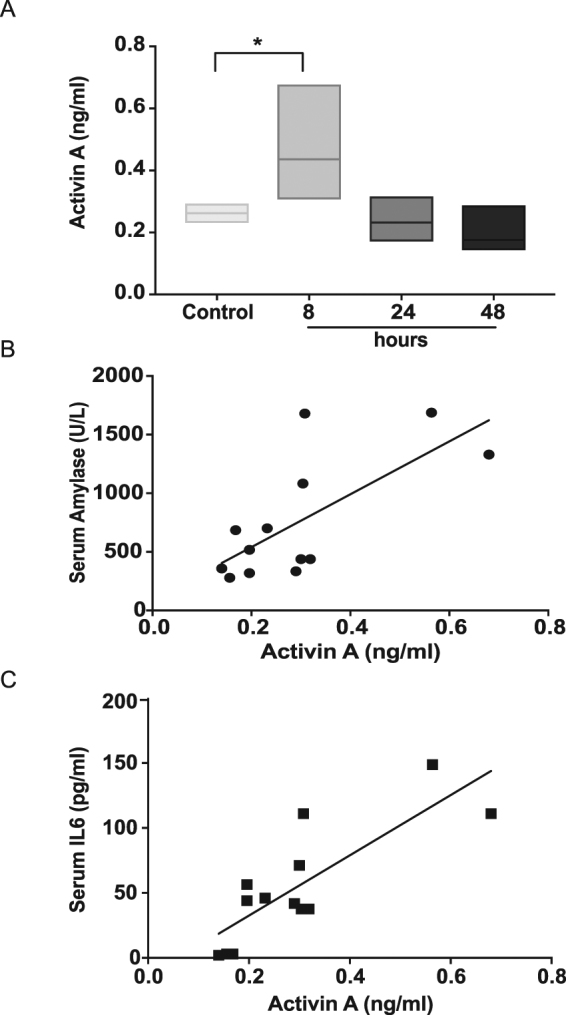
Activin levels are increased in a cerulein-induced model of acute pancreatitis. Panel A: Time course of cerulein treatment (hours) with activin serum levels obtained by ELISA and compared to vehicle treated controls as described in Methods. n = 4 for all groups except for 24 hours timepoint where n = 5, p = 0.026 per ANOVA with Dunnet’s post-test. Panel B: ELISA indicating serum amlyase (U/L) compared to activin levels for each cerulein-treated mouse. n = 13; r = 0.69, p < 0.05 per Pearson product-moment correlation coefficient. Panel C: ELISA of serum IL6 (pg/ml) compared to activin levels for each cerulein-treated mouse. n = 13, r = 0.818, p < 0.001 per Pearson product-moment correlation coefficient.

To investigate activin’s role in AP, we first investigated systemic activin levels in a standard, well-characterized murine model of AP, in which intraperitoneal (IP) cerulein peptide injections induce a mild edematous form of acute pancreatitis observed by changes in pancreatic histology (Supplementary Figure 1). We observed an approximately 2-fold increase in circulating activin ligand at 8 hours (average 0.5655 ng/ml versus 0.26 ng/ml; p = 0.026) when compared to the control group, but no change at 24 or 48 hours (Fig. 1A). Activin levels in animals with AP correlated strongly with circulating amylase (r = 0.69, p < 0.05), a marker for pancreatic tissue damage, and very strongly with IL-6 (r = 0.818, p < 0.001), a key component of the inflammatory response (Fig. 1B and C), supporting our initial hypothesis of a role for activin in AP.

### Circulating activin distinguishes severe from mild AP in a non-invasive model of severe AP

**Figure 2:**
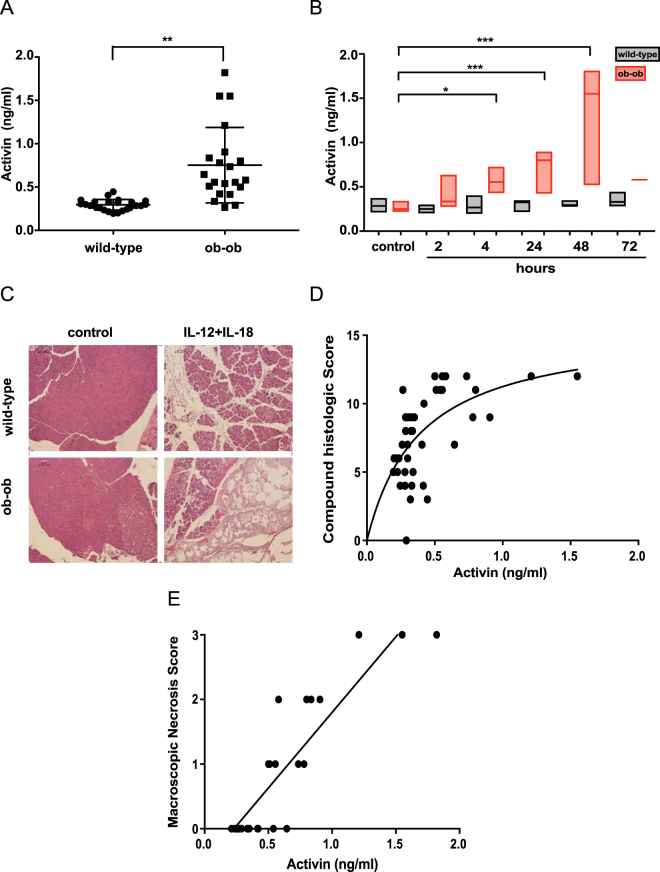
Activin levels are strongly increased in a model of severe necrotizing pancreatitis. Wild type and *ob*/*ob* animals were treated with IP injections of IL-12 + IL-18 on subsequent days as described in Methods, which leads to severe necrotizing pancreatitis in the *ob*/*ob* mice and mild pancreatitis in the control mice. Panel A: Serum activin levels measured by ELISA in wild-type compared to *ob*/*ob* mice (p < 0.01). n = 25 for wildtype and n = 21 for *ob*/*ob* animals, p < 0.05 per t-test Panel B: Serum activin levels in wild-type (gray bars) and *ob*/*ob* (red bars) at increasing time points. n = 5 per group except 72 hours in the *ob*/*ob* group where n = 1 due to mortality. ANOVA plus Dunnet’s post-test for statistical testing. Panel C: H/E stain of wild-type and *ob*/*ob* pancreas after 24 hours of IL-12 + IL-18 treatment. Panel D: Macroscopic necrosis score compared to activin serum levels. n = 46; r = 0.903, p < 0.0001 per Pearson product-moment correlation coefficient. Panel E: Compound histologic score compared to activin serum levels. n = 46; r = 0.5722, p < 0.001 per Pearson product-moment correlation coefficient.

As morbidity and mortality in AP primarily occurs in severe disease, we investigated activin in a murine model of severe necrotic pancreatic disease with mortality, mimicking severe AP in humans. Since activin is increased in animals after sham-operations^29,30^, we used a non-invasive induced model of AP, in which IP injections of IL-12 + IL-18 on subsequent days lead to aggressive necrotizing AP in *ob*/*ob* mice and a mild, edematous pancreatitis in wild-type animals. To verify the validity of the model, we histologically scored the pancreas for edema, lymphocytic infiltrate, acinar necrosis, and fat necrosis. As published previously^31^, the *ob*/*ob* animals displayed a more aggressive pancreatitis phenotype after IL-12 + IL-18 stimulation compared to wild-type animals (median histologic score 11/12 versus 6/12, p < 0.001). We then measured an array of cytokines critical in the inflammatory response in AP and observed cytokine patterns similar to those reported in human disease with marked increases in IL-6, IL-10, interferon-gamma and TNF confirming the applicability of our model. As anticipated^31^, cytokines were higher in severe disease when compared to mild disease (Supplementary Figure 2). Mice with severe disease also displayed a higher level of activin when compared to mild disease (Fig. 2A). We observed no change in systemic activin levels in mild AP compared to animals treated with vehicle control at any time point. However, as early as 4 hours (Fig. 2B), we observed marked and highly statistically significant increases in activin levels in severe AP. Activin levels also strongly correlated with macroscopic necrosis (r = 0.903, p < 0.0001) (Fig. 2D) and histologic severity score (r = 0.5722, p < 0.001) (Fig. 2E). Interestingly, of all histologic parameters scored, the correlation was strongest with histologic fat necrosis (r = 0.686, p < 0.001).

Next, we performed Receiver Operator Characteristic (ROC) analysis to determine whether the level of circulating activin may distinguish between mild and severe pancreatitis. Comparing all animals with mild and severe disease, respectively, activin proved to be an excellent marker for severe disease with an area under the curve (AUC) of 0.928. In summary, activin *in vivo* strongly correlates with disease severity and is an excellent marker to distinguish animals with mild and severe pancreatitis, respectively.

### Increased activin levels in severe pancreatitis are independent of mouse genotype and correlate with reported mortality rates

*Ob*/*ob* animals are leptin deficient due to homozygous mutation in the *ob* gene^32^. To confirm that increased activin levels in pancreatitis are independent of leptin deficiency, we used a complementary IL-12 + IL-18 induced model of necrotizing AP based on a high fat diet leading to diet-induced obesity (DIO). Animals with DIO develop severe pancreatitis, even though the phenotype is somewhat milder when compared to AP induced in *ob*/*ob* animals^33^. Induction of circulating activin was observed only in animals with DIO (Supplementary Figure 3), but not in control animals on normal chow. The increase in systemic activin levels correlated well with the mortality rates in our models of severe AP, which was reported to be approximately twice as high in the *ob*/*ob* mice (80–90%) when compared to the DIO animals (30–50%) and confirmed that the increase in activin levels in *ob*/*ob* mice with pancreatitis is independent of leptin.

### Continuing Activin inhibition conveys survival benefit in severe AP

Next, we explored whether inhibition of activin may lead to an improved outcome in severe AP. As the clinical challenge in treating AP is to reduce mortality in severe disease, we monitored mortality in a model of severe AP as an endpoint. Follistatin is a physiologic and specific antagonist that inhibits activin through trapping the ligand with high affinity^10,11^. Follistatin has previously been used to inhibit activin in murine models of IBD^34^ and endotoxemia^16^. One limitation to this approach is the short *in vivo* half-life (below 15 minutes^35^) of follistatin limiting bioavailability and the compound cost which limits the use of repeated dosage. Given these constraints, we assessed whether early administration may have any effect on ensuing mortality. Consistent with a causative role of activin in severe AP, we observed a biologically relevant and statistical significant, albeit modest, reduction of mortality on the first day of treatment (23% versus 0%, p < 0.05) with an overall favorable hazard ratio of 0.579, which did not reach statistical significance. (p = 0.3888, 95% CI 0.176 to 1.908) (Supplemental Fig. 4).

**Figure 3:**
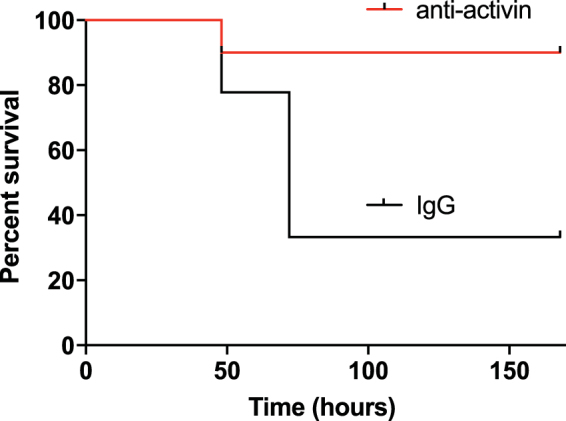
Activin inhibition through activin neutralizing antibody treatment is protective against mortality in acute pancreatitis. *Ob*/*ob* animals where pretreated for 30 minutes with either anti-activin antibody (red line, n = 10) or non-specific IgG (black line, n = 9) before administration of IL12 + IL18 and 24 hours after the last IL12 + IL-18 administration. Live animals were recorded on each day for one week. Hazard ratio 0.146 (0.028 to 0.7251) for anti-activin antibody versus non-specific IgG, p = 0.0189 per Mantel-Cox test.

In a second approach, we used a neutralizing antibody against activin which has been reported to block activin *in vivo* ^36^. The serum half-life of monoclonal IgG antibodies in adult mice is estimated to be 6 days^37^ which obviates the short half-life issue of the follistatin approach. In addition, preliminary experiments in pancreatic cancer tissue culture cell lines demonstrate that the activin neutralizing antibody blocks activin functions as measured by a decrease in SMAD2 phosphorylation (data not shown). Acute severe pancreatitis was induced by IL-12 + IL-18 injections in *ob*/*ob* mice as detailed above. The mice were divided into two arms, one receiving the activin neutralizing antibody (anti-activin) and the second arm receiving a non-specific IgG (IgG). The mice were monitored for mortality for 7 days. As shown in Fig. 3, the mortality in the anti-activin group was 1/10 while the mortality in the non-specific IgG group was 6/9 (Mantel-Cox p = 0.0189 and Hazard ration of 0.1426 (95% CI 0.028 to 0.7251) reducing the mortality from 66% to 10%.

### Serum activin is increased in AP, strongly correlates with severe pancreatitis, and is predictive of worse prognosis in patients with pancreatitis

**Figure 4:**
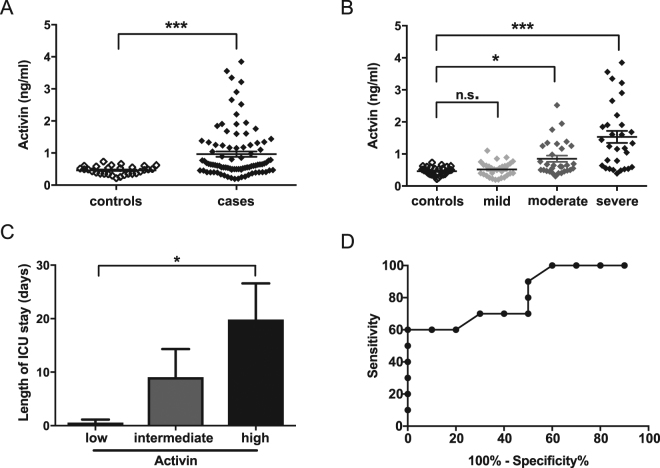
In patients with acute pancreatitis, activin is increased in moderate and severe disease and increased activin correlates with worse prognosis. Panel A: Serum collected from admission and two subsequent days was collected from each pancreatitis case in the cohort and serum activin measured by ELISA. Similarly, samples from one time point from healthy controls were collected and serum activin compared to pancreatitis cases. n = 30 for controls and n = 90 for cases, p < 0.001 per t-test. Panel B: Pancreatitis samples are grouped by severity as per revised Atlanta criteria (controls are open diamonds, mild are light gray diamonds, moderate are gray diamonds and severe pancreatitis are black diamonds). n = 30 per group, ANOVA plus Dunnet’s post-test used for statistical testing. Panel C: Cases were grouped by activin levels at admission with cut-offs at 25% and 75% percentile. Average length of ICU stay is shown for each group. n = 16 for intermediate and n = 7 for activin high and low groups. ANOVA plus Dunnet’s post-test used for statistical analysis. Panel D: ROC analysis for distinguishing mild versus severe pancreatitis using admission activin levels n = 20, AUC 0.820.

After these promising results in our animal models, we proceeded to investigate activin levels in a human cohort of AP, consisting of a total of 30 cases with 10 cases of mild, moderate and severe pancreatitis (as per revised Atlanta criteria^4^) respectively and 30 controls. Serum was collected as close to hospital admission as possible, and on each of two subsequent days. Overall, serum activin levels were increased in acute pancreatitis samples when compared to controls (0.965 ng/ml versus 0.462 ng/ml, p < 0.0001) (Fig. 4A). When grouped by severity, activin was specifically increased in moderate and severe AP, but not in mild disease (p < 0.0001 for difference in between groups, p < 0.05 for moderate versus controls, p < 0.0001 for severe versus controls, mild versus controls n.s.) (Fig. 4). This effect was seen both in samples at admission and comparing all samples from AP cases. Activin levels from subsequent blood draws were not statistically different from first activin measurements (Supplementary Figure 5). Importantly, high activin levels at admission were predictive of a longer hospital stay when compared to intermediate or low activin levels (median 26 versus 8 versus 5 days, p < 0.05, Supplementary Figure 6) and predicted a longer stay in the intensive care unit (ICU) (median 23 versus 0 versus 0 days, p < 0.05, Fig. 3). Also, activin levels at admission distinguished between mild and severe disease with an AUC of 0.8200 (Fig. 4), with an even higher predictive power (AUC of 0.8900) at the time of the second blood draw. As a control experiment to put our findings of activin in AP into context, we assessed circulating activin levels in a human IBD cohort. In contrast to the findings in our AP cohort, activin levels were unchanged from control in a spectrum of patients with IBD (Supplementary Figure 7, p = n.s. for difference between groups), with no correlation of activin serum levels with clinical inflammation activity. This clearly indicates that activin levels are not elevated in all inflammatory diseases, and that there may be a specific role of activin in acute pancreatitis.

## Discussion

This is the first study investigating activin levels in AP, in which we demonstrate that activin, a TGF-β superfamily member and key modulator of the inflammatory response, is upregulated in two distinct animal models and a human cohort of AP. It should be noted that activin ligand exists in various isoforms. There is a limited understanding of the potential differences in the function of these isoforms in the context of inflammation. Our study focused on activin A, the most common and best studied isoform in the context of inflammation^16,38^ and is referred to as activin throughout the manuscript.

To our knowledge, all preclinical model of AP have limitations^39^. To minimize those, we used two very distinct animal models of AP – a cerulein-induced standard model of mild AP, and an IL-12 + IL-18 induced model that leads to severe or mild AP depending on whether it is used in *ob*/*ob* or wild-type animals. The IL-12 + IL-18 induced model, extensively characterized in previous studies by our group^31,33^, is a clinically applicable, reproducible, non-invasive model of necrotizing AP. Even though neither model mimics AP physiology with regards to etiology, the reproducibility of our animal data in our human cohort supports the use of the models we chose. It should also be noted that we used animals from a balb/c background in our cerulein experiments and C57BL6 animals in our IL-12 + IL-18 driven models, which decreases the chance that our observations are background-dependent, albeit limiting direct comparison across models.

Our clinical cohort enabled us to investigate activin for the first time in AP to include outcome, yet limitations should be kept in mind. We analyzed this cohort retrospectively and biliary or post ERCP etiologies of AP are overrepresented when compared to other published cohorts, while patients with ETOH-induced pancreatitis are not included. These will need to be addressed in future prospective studies following our pilot study reported here.

Clinically, identifying patients that will transition to more severe disease is challenging^40^. Clearly, a treatment that minimizes the deleterious effects of severe inflammation without interfering with the normal healing process is needed, as it benefits patients directly and could markedly reduce health care costs. Existing predictive tools are limited and complicated by either a complex algorithm or the need for a second time-point, or both. A simple early predictive marker especially allowing stratification regarding hospital admission or need for ICU care would have a substantial impact on the management, outcomes of AP as well as the cost-effectiveness of its care.

In this pilot study, we observed a significant correlation of elevated activin ligand levels at admission with clinical severity in AP. These observations are very promising for stratifying AP patients, especially as high activin ligand was also predictive for longer hospital stay and higher rates of ICU admission. Whether activin can be combined with existing clinical markers to increase predictive power of current diagnostic criterion and if high activin levels are predictive for increased mortality will be addressed in future research directions.

In addition, activin showed promise as a potential therapeutic target in AP. Our experiments demonstrated that the activin inhibitor follistatin reduced mortality at an early time point in our model of severe AP. The observed overall hazard ratio for follistatin of 0.579 is encouraging and biologically relevant. Serum half-life of follistatin is less than fifteen minutes *in vivo* ^35^, which limits its efficacy over time as a single dose treatment. In a second approach we utilized an antibody which neutralizes activin to inhibit activin prior to onset of severe AP in the *ob*/*ob* mouse model. We observed a robust decrease in mortality compared to non-specific IgG control animals indicating that activin has a functional role in AP-induced mortality. Future experiments will address if blocking activin after the initiation of AP can lead to a decrease in mortality providing a potential therapeutic application.

Increased knowledge of the mechanism of activin action in inflammation in general and AP specifically and better pharmacologic inhibitors will help us to fully elucidate the potential of activin inhibition in severe AP. Given the well tolerated safety profile of activin inhibition in clinical phase II studies of cancer associated anemia and the lack of current therapeutic options in AP^41^, activin inhibition holds great promise as a therapeutic intervention in AP.

Activin levels have been described to be increased in other inflammatory conditions including sepsis^42,43^, cystic fibrosis^44^ and allergic airway disease^45^. The data obtained in our IBD cohort indicates that activin does not play a central role in all human inflammatory conditions. A close connection between activin and neutrophils has been proposed before^46^. Neutrophils play a major role in severe acute pancreatitis^47^, however, IBD is thought to be a process driven mainly by T-lymphocytes^48^. Our findings might hint towards a dominant role of activin in neutrophil-driven inflammation. Additional mechanistic studies will be needed to fully understand activin’s action on neutrophils. Moreover, the predominant underlying cause of mortality from AP is organ failure, which is caused by an uncontrolled self-sustained cytokine response. As serum activin is specifically increased in severe cases of AP, but unchanged in mild AP, we propose that activin plays a role in perpetuating the overshooting cytokine response, and may not directly be connected to AP etiology per se. This is consistent with our observation that activin levels remain at control levels in the IL-12 + IL-18 mild form of AP in wild type mice, while activin levels increase as early as 4 hours in the IL-12 + IL-18 severe form of AP in ob/ob mice. This notion would make activin a prime therapeutic target in a number of conditions such as multi-organ failure in sepsis.

In conclusion, serum activin is a novel marker for the prediction of severity and hospital course of AP, as well as a potential new therapeutic target in this poorly understood and highly morbid condition.

## Methods

### *In vivo* models of acute pancreatitis

All animals used in our study were obtained from The Jackson Laboratory.

#### Cerulein model of pancreatitis

For the cerulein model, female Balb/c mice, 8 to 10 weeks old, received 7 hourly injections of cerulein (R&D systems, Minneapolis MN, USA) at 50 micrograms/kg, while controls mice received PBS injections^49^.

#### IL-12 + IL-18 model of pancreatitis

For the IL-12 + IL-18 model, male C57BL6 mice were fed either regular chow or high-fat diet (diet induced obesity (DIO), 60% Kcal/fat from Research Diets, New Brunswick NJ) for 6 weeks and the experiments performed at 20 weeks of age. The mice on diet as well as the C57Bl6 female WT and *ob*/*ob* mice received two IP injections of a combination of IL-12 (150 ng/mouse) and IL-18 (750 ng/mouse) (R&D systems) 24 h apart, whereas control mice received PBS^31,33^. Cage randomization was used to assign animals to either treatment groups or time groups. Mice were euthanized at time points indicated in the figures after the end of treatments; blood and tissues were collected for analyses. To assess the effects of activin inhibition on survival, female *ob*/*ob* mice received an IP injection of human recombinant follistatin 288 (R&D systems) (10 micrograms/mouse) 30 minutes prior to each injection of IL-12 + IL-18. As a control, the mice received an equivalent injection of phosphate buffered saline alone. Survival was monitored for up to 8 days post induction of AP. As a second approach, female *ob*/*ob* mice (n = 10) received 5 mg/kg of an activin neutralizing antibody (AF388, R&D Systems) 30 minutes prior to the first IL-12 + IL-16 injection and 24 hours after the last IL-12 + IL-18 injection. This antibody was previously reported to effective in blocking activin *in vivo* ^36^. As a control, female *ob*/*ob* mice (n = 9) received injections of a similar concentration of a non-specific IgG. Mortality was monitored as described above. All samples and histologic assessment of AP were blinded and coded by sample identifier. Blinding was revealed after assessment for statistical analysis.

### Sample processing and histologic assessment of AP

Murine serum samples were allowed to clot at room temperature for 30 minutes, centrifuged and aliquoted. Samples were subsequently stored at −80 degrees. Pancreas, liver, and lung tissue was fixed in 10% formalin, paraffin-embedded, and sectioned at 4mm interval as previously described^50^. Macroscopic necrosis was scored as previously described. In short, severity of necrosis was scored as 0 (absent), 1 (few pinhead-sized necrotic areas without retropancreatic necrosis), 2 (moderately extended necrotic areas with moderate/extensive retropancreatic necrosis), and 3 (extensive areas of necrosis with extensive retropancreatic necrosis)^51^. Slides of the pancreas where then scored blindly for edema, inflammatory infiltrate as well as acinar and fat necrosis using a previously reported score^31^.

### Quantification of cytokine serum levels

Activin from human and murine samples was measured utilizing the activin A Quantikine ELISA (R&D Systems) following the manufacturer’s instructions. All samples were run in duplicates after a 1:4 dilution in PBS. A custom multiplex assay for interferon-gamma, TNF, IL-6 and IL-10 was purchased from R&D Systems and run following the manufacturer’s instructions. All samples were run in duplicates.

### Human pancreatitis cohort

Our retrospective cohort consisted of a total of 30 cases with 10 cases of mild, moderate and severe pancreatitis respectively (as per revised Atlanta criteria^4^) and 30 controls. Under IRB approved protocols at the University of Pittsburgh, serum was collected as close to hospital admission as possible, and on each of two subsequent days. Throughout the subject’s hospital course, blood was collected daily on day 1 and, while the patient remained in the hospital and sampling was possible daily through day 7, and then weekly (starting on day 14 + /− 1 day), through day 28 or until discharge whichever comes first, for noting the trend in inflammatory markers and in enzymes of pancreatic injury.

Clinical information required for severity assessment using the Revised Atlanta Classification criteria^4^ and for determination of the etiology of acute pancreatitis was obtained from patient’s medical records and by following the hospital course. The necessary information includes clinical parameters, routine laboratory tests, imaging results, treatment interventions and other outcome variables. The clinical cohort was established previously using clinical images reviewed for the research study by UPMC radiologist co-investigators to assess for presence of any remote organ complications important in defining complications of acute pancreatitis. Data elements were collected using standardized case report forms and entered into a secure database on initial admission and on subsequent days of hospitalization. Subjects were divided into mild, moderate and severe acute pancreatitis following the Revised Atlanta Classification system^4^.

For each subject recruited, approximately one unrelated family member who did not have a history of pancreatitis was recruited as a control. In cases where there was no such control available or willing to participate, we recruited the control from general medicine outpatient clinic when he/she needed to have the blood sample drawn for other reasons or was willing to provide the blood sample for the research purpose. This provides for an unrelated control population and a balanced estimate of allele frequencies^52^. We matched the subjects and controls for ethnicity and sex.

### Human Inflammatory Bowel Cohort

We received serum from a subset of the Regensburg IBD cohort consisting of 45 patients with Crohn’s Disease (CD) and 46 patients with ulcerative colitis (UC). Details of this cohort have been published previously^53–56^. Both the CD and UC arms of the cohort were sub-divided into 3 categories of 15 patients each based on the state of the disease at the time of collection; namely active, chronic active or in remission according to the Vienna Classification and CD activity index (CDAI) for CD and the Truelove-Witts index for UC.

### Statistical analysis

Data are expressed as mean ± SD for continuous variables, median and range for categorical variables, respectively. Statistical significance level of α = 0.05 was set before experiments. All statistical tests were two-sided if not noted otherwise. For correlation analysis, a Pearson product-moment correlation coefficient was used. For comparison of three or more groups, one-way analysis of variance (ANOVA) test with Dunnet’s post-test was used to test differences among groups and adjust multiple comparisons of each experiment group with a single control. Considering the two samples under testing have unequal variances and unequal sample sizes, for comparison of two groups, a two sided Welch’s unequal variances *t*-test was utilized. The modifiers strong and very strong with regards to correlations as referred by the statistical significance of calculated R values is per published methodology^57^.

To determine predictive power of activin, we performed receiver operator characteristic analysis and calculated the respective area under the curves (AUC). For overall survival and mortality at day one analysis, Mantel-Cox and one sided Barnard’s test were utilized, respectively. For comparison of the rate of ICU admission in our clinical cohort, we used Fishers exact test with Freeman-Halton extension. Differences in activin levels at the different time points was investigated using a repeated measure ANOVA. All statistical analyses were performed using GraphPad Prism version 5.00 for Windows (GraphPad Software, San Diego CA).

### Study approval

All experiments involving animals were approved by the Animal Care and Use Committee (ACUC) of the University of Illinois at Chicago and all methods were performed in accordance with the guidelines and regulations of the ACUC. Acute pancreatitis and normal control subjects were recruited into this retrospective study at University of Pittsburgh Medical Center (UPMC) sites and at the Veterans Administration (VA) Pittsburgh under protocols approved under the University of Pittsburgh Institutional Review Board (IRB). Informed consent was received for all participants prior to their inclusion in the study. The subject serum was provided to the University of Illinois Chicago as coded by number and de-identified to remove all private health information. The serum was analyzed at the University of Illinois at Chicago after determination of non-human subject research status by Institutional Review Board. Inflammatory bowel human cohort subjects were recruited under institutional approval at University of Regensburg, Regensburg, Germany and the serum analyzed at the University of Illinois at Chicago as detailed above. All human studies were performed in accordance with the guidelines and regulations of their respective institutions.

### Data Availability

All data generated or analyzed during this study are included in this published article (and its Supplementary Information files).

## Electronic supplementary material

Supplementary Figures
