# Supplementary material for: Activin in acute pancreatitis: Potential risk-stratifying marker and novel therapeutic target
Source: Sci Rep. 2017 Oct 6;7:12786. doi: 10.1038/s41598-017-13000-3 (PMC5630611; doi:10.1038/s41598-017-13000-3)
Supplement: Supplementary file 1 — Supplementary Figures [file 41598_2017_13000_MOESM1_ESM.pdf]

Supplementary figures for: Activin in acute pancreatitis: Potential risk-stratifying marker and novel therapeutic target

Authors: Jonas J. Staudacher[1], Cemal Yazici[1], Timothy Carroll[1], Jessica Bauer[1], Jingbo Pang[2], Nancy Krett[1], Yinglin Xia[1], Annette Wilson[3], Georgios Papachristou[3,4], Andrea Dirmeier[5], Claudia Kunst[5], David C. Whitcomb[3], Giamila Fantuzzi[2] and Barbara Jung*[1]

Supplementary Figure 1

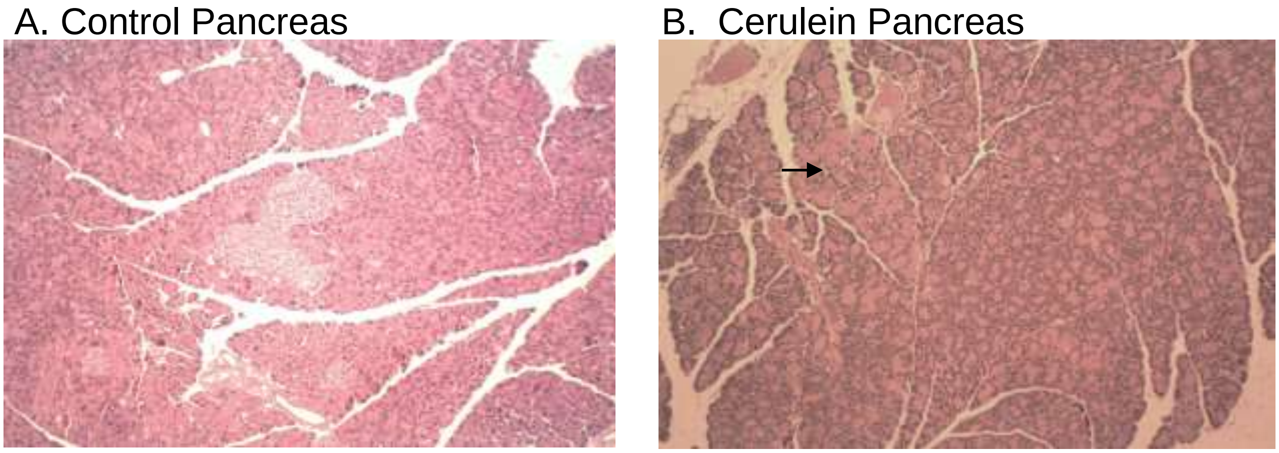

Figure S1. Cerulein induces mild pancreatitis at 8 hours with evidence typical histologic changes. Hematoxylin and eosin staining of representative mouse pancreas after 8 hours of either control treatment (panel A) or cerulein treatment (panel B) with 10X magnification. The slides were scored blinded by a trained pathologist and shown in the reflective of the gradual change seen with moderate pancreatitis. The arrow indicates reduced zymogen integrity preceding pancreatitis in the cerulein treated mice.

Supplementary Figure 2

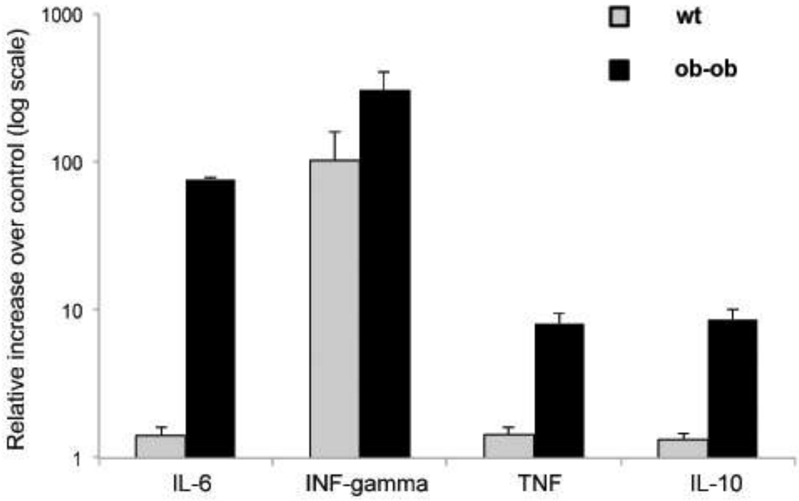

Figure S2. Serum cytokines in IL-12 + IL-18 induced model of severe (ob/ob) and mild (wild-type) pancreatitis. The cytokines indicated in the X-axis were measure by multiplex assay as described in Methods. Relative increase at 24 hours compared to control animals is shown in log scale. These data confirm the previously described model (28). n=5 for all groups. Significance for each cytokine is a follows: IL-6, p=0.0094; INF-gamma, p=0.0952; TNF, p=0.0317; IL-10, p=0.0362. The two-tailed Mann-Whitney test was used for statistical analysis.

Supplementary Figure 3

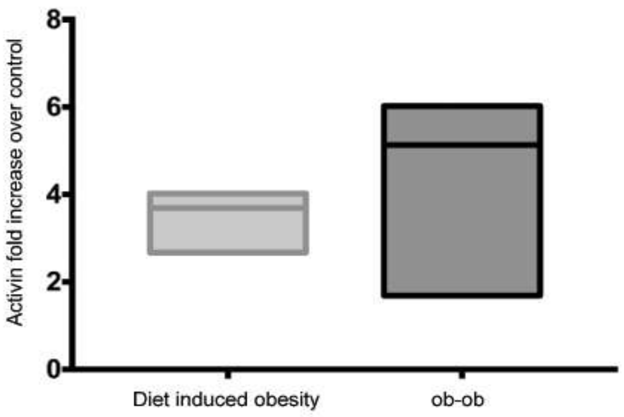

Figure S3. Increase in serum activin in *in vivo* model of severe pancreatitis is not dependent on ob genotype. Serum activin levels were measured by ELISA in diet-induced obese animals compared to ob/ob animals after induction of severe pancreatitis by IL-12 +IL-18 treatment. The fold increase relative to control 24 hours after second injection is depicted. n=5 for ob/ob and n=3 for diet induced obese model.

Supplementary Figure 4.

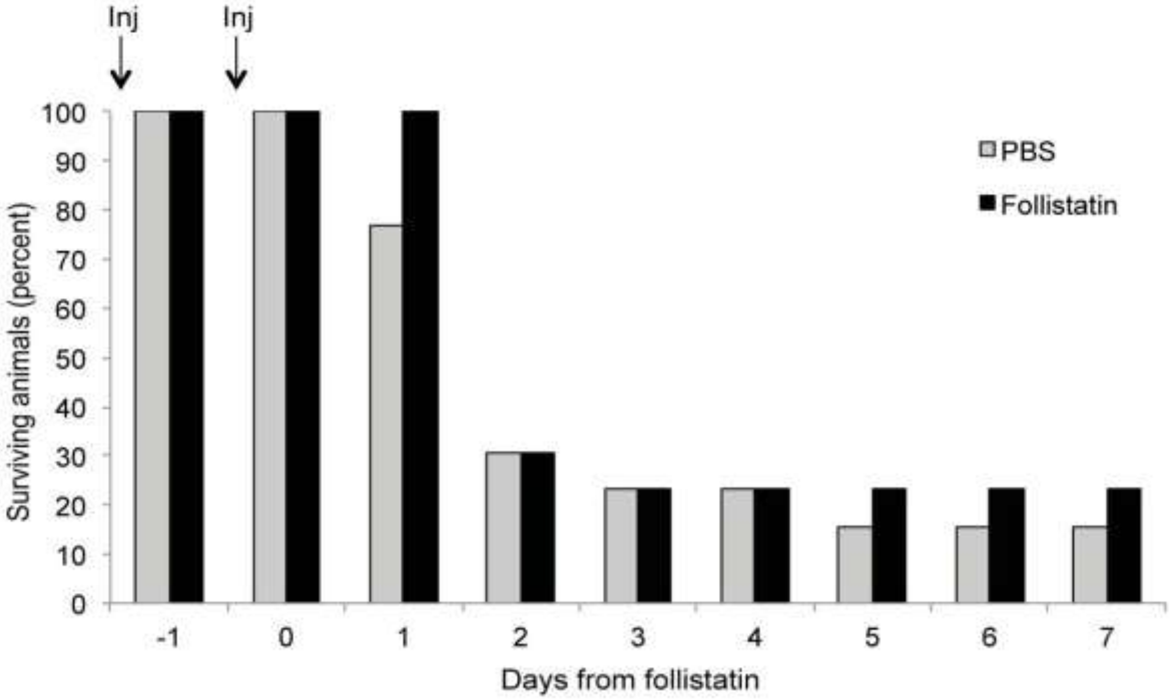

Figure S4: Activin inhibition through follistatin treatment is protective against mortality in acute pancreatitis. *Ob/ob* animals where pretreated for 30 minutes with either follistatin (black bars) or PBS (gray bar) before administration of IL12+IL18 (arrows indicate times of first and second injection). Live animals were recorded on each day for one week. n=13 per group, Hazard ratio 0.579 for follistatin versus PBS, p=0.38 per Mantel-Cox test.

Supplementary Figure 5

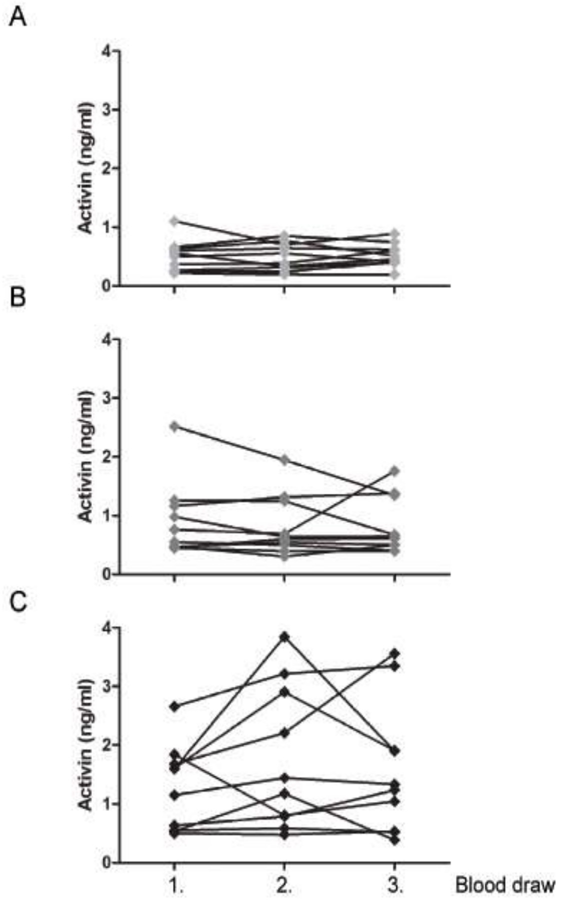

Figure S5. Serum activin levels in pancreatitis cohort at subsequent blood draws do not significantly differ from blood draw at admission. Serum activin levels at first, second and third blood draw are depicted. Panel A: Mild pancreatitis patients; Panel B: Moderate pancreatitis patient and Panel C: Severe pancreatitis patients. n=10 per severity group and time point, total n=90.

Supplementary Figure 6

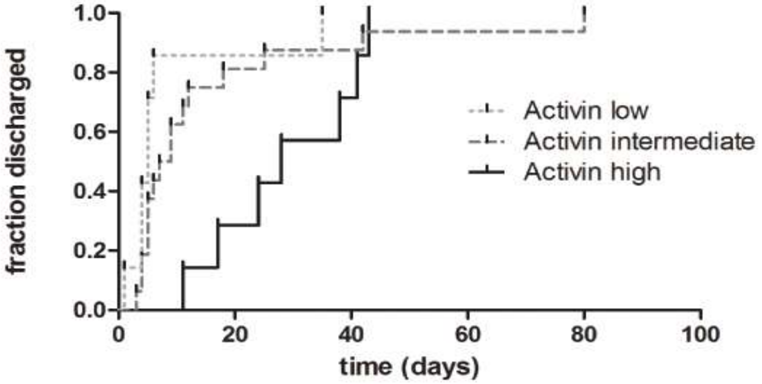

Figure S6. Activin levels at admission are predictive of longer overall hospital stay. Patients were group by serum activin levels at admission. Activin low is lower than 25% of serum activin in cases in our cohort; Activin intermediate is between 25th and 75th percentile; and activin high is above 75th percentile. Data are plotted as Kaplan Meier curves comparing fraction discharged to time of hospital stay. n=16 for activin intermediate and n=7 for activin high and low groups.

Supplementary Figure 7

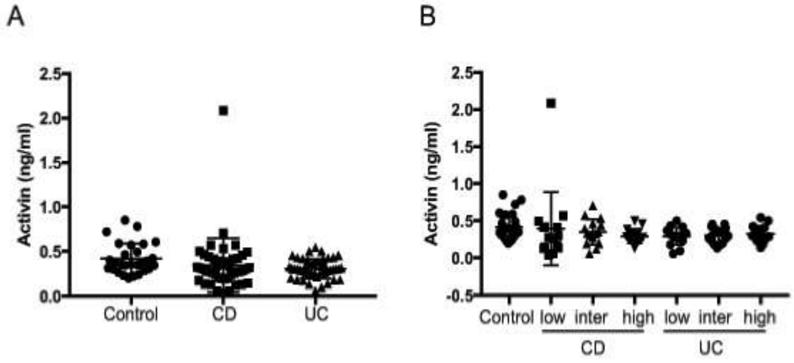

Figure S7. Activin is not increased in patients with inflammatory bowel disease (IBD). **A**: Activin measured by ELISA assay in the serum from each IBD case in the cohort. Similarly, serum from healthy controls was collected and activin measured and compared to IBD cases. n=28 for control, n=45 for Crohn's disease (CD) and n=46 for ulcerative colitis (UC). **B**: IBD samples are grouped by severity as low (remission), intermediate (chronic active), and high (active). n=15 each group except UC intermediate n=16.
